# Revolutionizing Educational Assessment: The Role of Bing Chat GPT-4 Chatbot in Increasing Efficiency in Grading Open-Ended Questions

**DOI:** 10.7759/cureus.100508

**Published:** 2025-12-31

**Authors:** Diane Abadam-Eremeev, Rachel P Seng, Janani Srinivasan, Kyle Knouna, Joseph R Bogaudo, Paulo A Rodriguez De La Nuez, Alexey Podcheko

**Affiliations:** 1 Research, American University of the Caribbean, Cupecoy, SXM; 2 Endocrinology, American University of the Caribbean, Cupecoy, SXM

**Keywords:** artificial intelligence and education, automated grading, chatgpt, deep learning, generative ai, medical education, open-ended questions, recall memory

## Abstract

Background

Bing Chat (Microsoft Corporation, Redmond, WA) is an artificial intelligence (AI) program that can respond to typed text. Many educational institutions are exploring the incorporation of AI into their materials, given its rapid advancements and potential to streamline various processes. One area that still requires investigation is the ability of AI models to grade open-ended questions. The purpose of this study is to determine whether Bing Chat or university faculty exhibit greater consistency in grading such questions.

Methods

The authors recruited 21 medical students from the American University of the Caribbean (AUC) to answer five open-ended questions related to the United States Medical Licensing Examination (USMLE) Step 1 topics. The volunteer participants consisted of first-year and second-year medical students. The responses of each student to the five questions were graded by six different Bing Chat accounts and six faculty members. Differences in scores between Bing Chat and the faculty members were compared, and inter-rater reliability estimates were calculated.

Results

Both Bing Chat and faculty consistently measured the same responses; although there was some variability in both cases, it was more pronounced in faculty grading. For analysis, problem-solving questions with elements of application, explanatory, and recall-type questions, Bing Chat’s grading closely paralleled that of the faculty. However, when grading a combined recall-and-application question, a significant gap between Bing Chat and faculty scores was observed (p = 0.010). Overall, Bing Chat demonstrated higher inter-rater reliability than faculty, as evidenced by both percent agreement and Gwet's agreement coefficient 1 (AC1).

Conclusion

Bing Chat demonstrated promising results in evaluating written answers to open-ended questions and shows potential as a supportive grading tool. As educational leaders seek more dependable, faster, and economical methods for assessment, Bing Chat may offer a notable contribution to education. Large language models (LLMs), such as Bing Chat, can be beneficial to both students and educators.

## Introduction

Medical schools are continuously evolving their methods to effectively assist students in succeeding in their medical education. They are striving to find ways for students to excel in passing the United States Medical Licensing Examination (USMLE). The quest for a game-changing innovation in educating medical students continues. Although multiple-choice questions, an example of recognition memory, are most widely used for testing due to the convenience of grading as well as the standardization of responses, prior studies have shown that the best way to solidify learning is by using recall memory [1-4].

Open-ended questions challenge students’ recall memory and have proven to be the most effective way to solidify learning [3-5]. When responding to open-ended questions, students provide text answers in either a natural or formal language, enabling a more in-depth assessment of their capabilities and learning performance [1,6]. However, it is important to note that assessing these responses can be time-intensive, demanding significant concentration, and may also be influenced by the subjectivity of graders [1,6]. Evidence has established that artificial intelligence, such as ChatGPT (OpenAI, San Francisco, CA), is a useful tool that has sparked research in every scientific field due to its vast database of knowledge [7,8].

Students’ learning is enhanced when they can answer open-ended questions compared to multiple-choice questions [1,4,5]. Recall is believed to be much stronger for long-term memory than recognition [3,5,6]. If AI models can grade and provide accurate feedback to open-ended questions that are comparable or better than the assessments done by the faculty, then the education industry would benefit by more widely implementing AI models and open-ended questions into their programs as a component of formative or summative assessments. This study tests the limits of Bing Chat (Microsoft Corporation, Redmond, WA) by examining its accuracy in grading open-ended responses in comparison to evaluations provided by the faculty (considered the gold standard), with the goal of enhancing students’ learning and alleviating educators' burden without compromising efficiency.

## Materials and methods

Data collection

The participants in this study were students at the American University of the Caribbean (AUC), in their first (15 students) and second (six students) pre-clinical year of medical studies. Details regarding the age of the students and sex were not collected. Informed consent was obtained from each participant. The study selected five open-ended questions curated to USMLE Step 1 topics. These questions are provided in Table 1. Faculty experts in the area related to the specific question provided the “Expert Answer” (Appendix A).

**Table 1:** Five open-ended questions and their domains.

Question number	Question	Question domain
1	A cell biologist is interested in the role of ribonucleoproteins in cellular processes. He isolates a specific type of cells from a human female donor who is 60 years old and extracts the ribonucleoproteins from them. He then performs structural and functional analyses on the purified ribonucleoproteins. He discovers that these cells have a higher expression of a protein that can synthesize TTAGGG repeats at the ends of chromosomes using reverse transcription. Explain what kind of cells he most likely used in his experiment and why.	Analysis
2	A 40-year-old man presents to the emergency department complaining of severe shortness of breath. The breathlessness has been worsening over the past few years, and the patient reports growing tachypneic with mild exertion, and sometimes even at night. On examination, he has generalized edema, jugular venous distention, and hepatic distention. Cardiac examination shows a right ventricular heave, a right-sided S3, and S4 with a pulmonary ejection click. A chest X-ray film shows cardiomegaly and widening of the hilar vessels, including the pulmonary arteries. An electrocardiogram shows tall, peaked P waves in leads lI, III, and aVF, right axis deviation, and right ventricular hypertrophy. Which physiologic stimuli will result in decreased pulmonary vascular resistance in this patient?	Problem-solving question with elements of application
3	Pulmonary hypertension is suspected in a 40-year-old patient with dyspnea, and a Swan-Ganz catheter is placed. Provide the correct anatomic sequence of vessels that would be traversed by the catheter if it were introduced into the left subclavian vein (start with the left subclavian vein)?	Recall and application
4	What is the physiological cause of red cell sickling in sickle cell disease?	Explanatory
5	Write down at least three notable signs or symptoms classically associated with polycythemia vera.	Recall question

An anonymous online questionnaire created via Google Forms (Google, Mountain View, CA) was distributed to 250 students, requesting that they respond to the open-ended questions to the best of their knowledge without utilizing any external resources (Appendix C). Students were informed that spelling or grammar was not evaluated, and the questions required written responses with no time restrictions. Students who completed the survey had a chance to enter a $100 gift card raffle, should they choose to participate. Responses were collected over one week, and 21 student responses were received (response rate of 8.4%). Participation in the study was voluntary, and student answers contained no personally identifiable information.

Analyses of responses

Bing Chat was used to evaluate each student’s response. To determine variability in the assessment of the same response by Bing Chat, six independent Microsoft Edge (Microsoft Corporation, Redmond, WA) accounts running Bing Chat were accessed on six different laptops/workstations: five using the Windows operating system (OS) and one using the Macintosh OS (macOS). Bing Chat settings were configured to “More Precise" conversation style, allowing for greater character allowance in the chat box. A standardized prompt was submitted to each device within one week following the collection of responses (Table 2). All student responses to question 1 were entered into Bing Chat using the designated prompt below. A new Bing Chat conversation was initiated for each response to be evaluated. Prompts for other questions followed a similar structure; for more details on prompt composition, refer to Appendix B.

**Table 2:** Standardized prompt entered into Microsoft Edge accounts running Bing Chat GPT-4.

Prompt
Please provide a concise response to the question/prompt below. Your answer will be graded based on accuracy and completeness. Use the following scale to assess each response:
5: The answer is complete. All information provided is accurate.
4: The answer is missing 2 or fewer details. All information provided is accurate.
3: The answer is missing multiple (>2) details. Most information provided is accurate.
2: Content suggests a lack of preparation or comprehension. Some information provided is accurate.
1: Content is only marginally related to the question/prompt. A small amount of the information is accurate.
0: Content fails to meet the basic requirements of the task. None of the information provided is accurate.
Below is the question we are going to be testing with the best answer provided (given a grade of 5 of 5):
A cell biologist is interested in the role of ribonucleoproteins in cellular processes. He isolates a specific type of cells from a human female donor who is 60 years old and extracts the ribonucleoproteins from them. He then performs structural and functional analyses on the purified ribonucleoproteins. He discovers that these cells have a higher expression of a protein that can synthesize TTAGGG repeats at the ends of chromosomes using reverse transcription. Explain what kind of cells he most likely used in his experiment and why?
Expert answer: Telomerase is a ribonucleoprotein that adds TTAGGG repeats to the 3' end of chromosomes (telomere region). Stem cells have very long telomeres due to their high telomerase activity, allowing them to proliferate indefinitely in a controlled manner. In contrast, most terminally differentiated adult somatic cells (e.g., myocardial cells, neurons, pancreatic β cells) have short telomeres as they do not express telomerase and their telomeres shorten with every cell division. The epidermis is continuously replaced by stem cells present in the basal cell layers. Bone marrow stem cells similarly replace peripheral red and white blood cells. Stem cells have long telomeres due to high telomerase activity, allowing them to proliferate indefinitely in a controlled manner.
Grade the following student response based on the grading criteria and the expert answer: (Student 1 response to question 1)

For comparison, six AUC medical faculty members also assessed the responses from 21 students. They had no access to student identities while grading, and they were given the same correct answers and the same standard rubric as the one given to Bing Chat. The standard rubric applies to all questions, outlining the expected level of detail for students to attain each of the five score levels (Table 3). The medical faculty was not involved in the selection of the five questions or the collection of data from Bing Chat.

**Table 3:** Standard grading rubric used to grade five open-ended questions by both Bing Chat and faculty.

Score	Score description
5	The answer is complete. All information provided is accurate.
4	The answer is missing 2 or fewer details. All information provided is accurate.
3	The answer is missing multiple (>2) details. Most information provided is accurate.
2	Content suggests a lack of preparation or comprehension. Some information provided is accurate.
1	Content is only marginally related to the question/prompt. A small amount of the information is accurate.
0	Content fails to meet the basic requirements of the task. None of the information provided is accurate.

A comparison was made between the grades provided by Bing Chat and those provided by medical school faculty. The hypothesis of this study is that, when provided with a grading rubric, Bing Chat will evaluate student responses to open-ended questions more consistently than medical faculty. The null hypothesis was the following: there is no significant difference in the grading scores between Bing Chat and the medical faculty.

Data analysis

To assess the consistency (i.e., the uniformity of scores among Bing Chat accounts) of grading the same answer by Bing Chat when the same prompt is submitted from different accounts, raw scores for each question provided by six Bing Chat accounts for 21 students were analyzed using line graphs. Similarly, the consistency (i.e., the uniformity of scores among faculty members) of grading the same answer by the faculty was assessed by plotting the raw scores that each faculty member gave to each individual for each question on a graph, resulting in line graphs. Then, the average of all Bing Chat and faculty scores for each of the 21 responses to each individual question was calculated and plotted on a bar graph. The comparison of the averages and trends in scoring for each group was analyzed. The purpose of these averages was to highlight the minor variations both within and between Bing Chat and faculty grading.

Additionally, the cumulative average score given by Bing Chat and faculty for all 21 responses combined for each individual question was calculated to compare, from a broader and simplified perspective, the differences between Bing Chat and faculty grading. ​Moreover, the average of Bing Chat and faculty scores for each respondent across all five questions together was analyzed to obtain a broader perspective of the potential variation of the scores. A graph showing the average comparison between Bing Chat and faculty grading for all responses across all five questions was created. Statistical tests for significant differences between the mean scores provided by Bing Chat and faculty were calculated using an unpaired t-test from GraphPad (GraphPad Software, San Diego, CA) [9]. A 95% confidence level (p < 0.05) was taken as the criterion of significance.

Inter-rater reliability study

The study further explores more rigorous statistical analysis by employing AgreeSTAT360 [10]. AgreeSTAT360 performs advanced statistical analysis of the extent of agreement among multiple raters [10]. The level of agreement between Bing Chat and faculty was assessed by examining their inter-rater reliability. Although there are several methods that AgreeSTAT360 uses to calculate the inter-rater reliability, this study mainly focuses on using percent agreement and Gwet's agreement coefficient 1 (AC1) methods to study inter-rater reliability between Bing Chat and faculty. Percent agreement was employed in this study because it highlights the concept of inter-rater reliability at its most basic level, simply put as the average amount of agreement expressed as a percentage [11]. Gwet's AC1, a more sophisticated inter-rater reliability method, was also employed, which avoids a number of situations where agreement is not adequately reflected by the measurement [12].

The extent of agreement among multiple raters (N = 6) was studied by computing percent agreement and Gwet's AC1 coefficients. The hypothesis was that there would be more agreement among Bing Chat-generated scores than among those assigned by faculty members. AgreeSTAT360 was used to analyze raw scores from six raters, using the chance-corrected agreement coefficients (CAC) method. Percent agreement and Gwet's AC1 coefficients were calculated for each student by entering the raw scores from six graders (either six Bing Chat accounts or six faculty members) as columns, corresponding to responses to questions 1 through 5 as rows (Table 4). This process was repeated for all 21 students. The average percent agreement and Gwet's AC1 coefficients were then computed, and statistical analyses were performed using the unpaired t-test calculator from GraphPad [9].

**Table 4:** For illustrative purposes only. Data entered into AgreeStat360 for analyzing raw scores to calculate the percent agreement and Gwet's agreement coefficient 1 (AC1) inter-rater reliability coefficients. The top columns represent the six raters, and rows correspond to the scored responses from student 1 for questions 1 through 5.

Bing Chat 1	Bing Chat 2	Bing Chat 3	Bing Chat 4	Bing Chat 5	Bing Chat 6
2	2	2	2	2	2
0	1	1	1	1	1
1	1	1	1	1	1
1	2	1	1	1	1
2	3	3	3	2	2

In addition, percent agreement and Gwet’s AC1 coefficients for each group were calculated per question. Raw scores from six raters evaluating the responses of 21 students for question 1 were entered in AgreeSTAT360 (Table 5). This process was repeated for questions 2 through 5 for each group. AgreeSTAT360 generated a table of inter-rater reliability coefficients across multiple methods (Table 6). Only data for percent agreement and Gwet's AC1 were used in this study. Graphs were developed to visualize and analyze the results. To assess whether the difference in Gwet's AC1 coefficients between Bing Chat and the faculty was statistically significant for each question, unpaired t-tests were conducted using AgreeTest software [13]. Statistical significance for the difference in percent agreement coefficients between Bing Chat and faculty was evaluated using Gwet's principle for correlated ratings and a conservative z-test [14]. Statistical significance was set at p < 0.05.

**Table 5:** For illustrative purposes only. Raw scores entered into AgreeStat360 were used to calculate the percent agreement and Gwet's agreement coefficient 1 (AC1) inter-rater reliability coefficients. The top columns represent the six raters, and the rows correspond to the scored responses from 21 students for question 1 only.

	Question 1					
Student	Bing Chat 1	Bing Chat 2	Bing Chat 3	Bing Chat 4	Bing Chat 5	Bing Chat 6
1	2	2	2	2	2	2
2	2	3	3	3	3	3
3	0	0	0	0	0	0
4	4	4	4	4	4	4
5	0	1	0	1	1	0
6	2	3	3	1	3	2
7	2	4	4	4	4	2
8	2	2	3	3	2	2
9	2	1	2	0	2	2
10	2	2	1	2	2	1
11	3	4	3	4	3	3
12	2	3	4	2	3	2
13	4	4	4	4	4	4
14	3	3	4	3	4	2
15	4	4	4	3	4	4
16	0	2	1	1	2	0
17	0	2	1	2	2	0
18	2	3	2	2	3	3
19	3	4	3	3	3	4
20	2	3	3	2	3	3
21	4	4	3	3	3	3

**Table 6:** For illustrative purposes only. Results from AgreeStat360 based on six raters evaluating 21 student responses to question 1, following execution of the inter-rater reliability test. Percent agreement and agreement coefficient 1 (AC1) data were used. Coeff: coefficient; StdErr: standard error; CI: confidence interval.

Inter-rater reliability coefficients and associated precision measures				
Method	Coeff	StdErr	95% CI	p-value
Conger's kappa	0.383	0.077	(0.222, 0.544)	3.793e-05
AC1	0.416	0.071	(0.268, 0.564)	4.909e-06
Fleiss' kappa	0.379	0.079	(0.215, 0.543)	5.255e-05
Krippendorff's alpha	0.384	0.079	(0.22, 0.548)	4.547e-05
Brennan-Prediger	0.409	0.072	(0.259, 0.559)	7.154e-06
Percent agreement	0.527	0.057	(0.407, 0.647)	6.603e-09

Ethics

This study received a letter of approval from the American University of the Caribbean, International Review Board (AUC IRB) on July 7, 2024.

## Results

**Figure 1:**
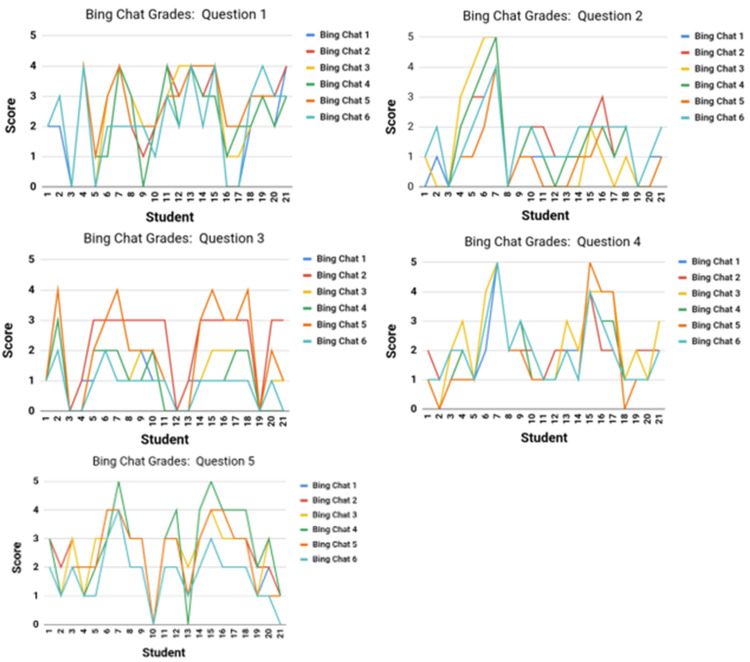
A summary of the Bing Chat-assigned scores for 21 students per question.

To compare the overall results between Bing Chat and faculty gradings, four questions (QI, QII, QIII, and QIV) were analyzed separately (Table 7). For question I (QI), the analysis focused on the consistency of Bing Chat's grading when the same prompts were submitted from six different user accounts. Graphs for each question were generated using the individual scores assigned by Bing Chat to 21 student responses. This analysis aimed to evaluate the consistency of Bing Chat's grading while accounting for different user accounts. With the exception of minor variations, the overall results across the responses and accounts appeared consistent (Figure 1).

**Table 7:** Study questions and analytical focus.

	Questions to analyze
QI	How consistent is the grading of the same answer by Bing Chat if the same prompt is submitted from different accounts?
QII	How consistent is the grading of the same answer across the faculty?​
QIII	Are there any differences in the grading of responses between Bing Chat and faculty based on the type of question categorized in Table 1?
QIV	How different are the grades assigned by Bing Chat vs. the faculty for each respondent across all five questions?​

**Figure 2:**
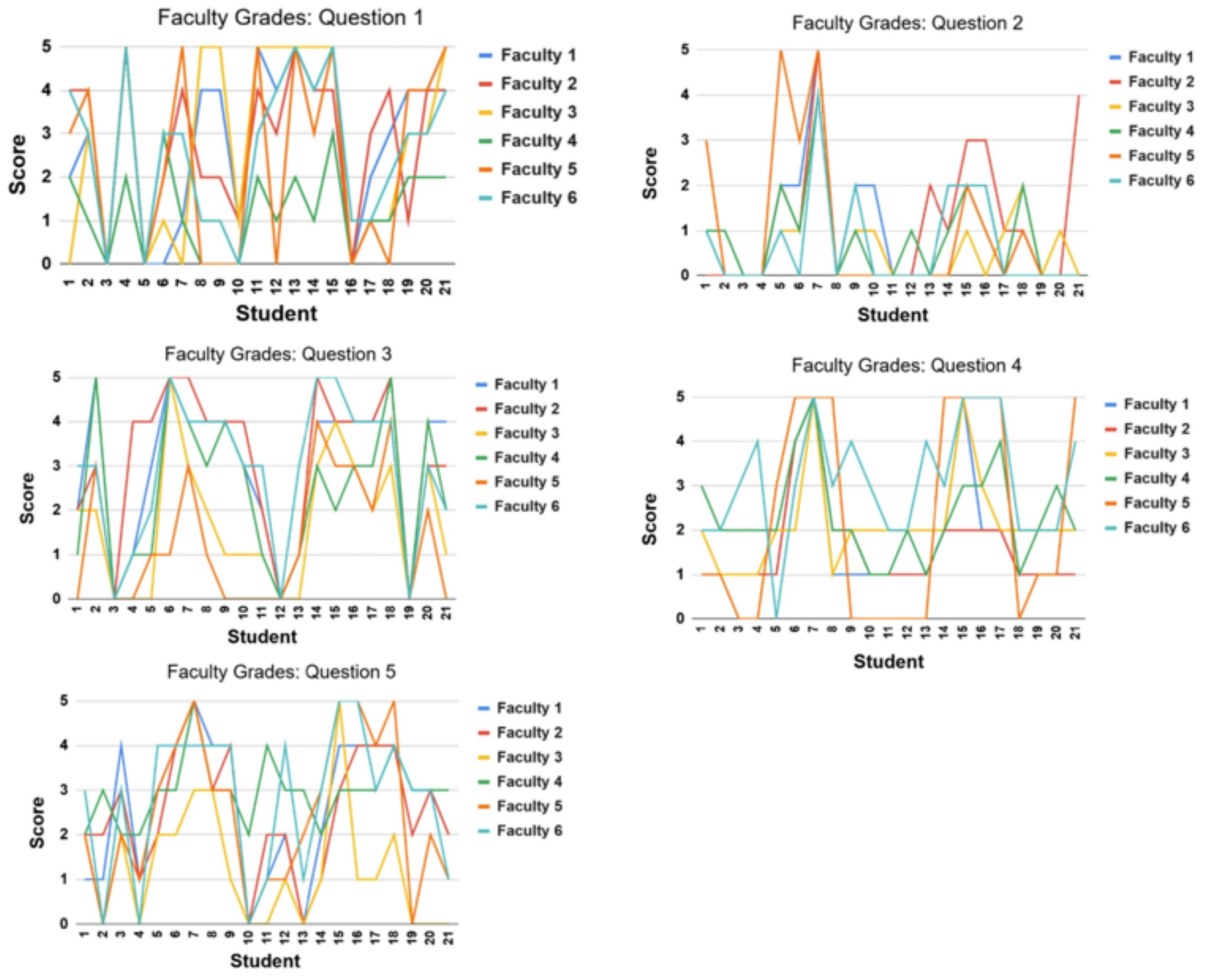
A summary of the faculty-assigned scores for 21 students per question.

Similarly, question II (QII) examines the consistency of faculty grading when evaluating identical student responses. To assess this, graphs were generated by using the scores assigned by each faculty member to 21 student responses to each question. These visualizations highlight the degree of variation among the faculty when grading the same response. Interestingly, comparison of Figures 1, 2 suggests that there is more variation among faculty members than in Bing Chat's assessments.

After analyzing the results of the scores provided by Bing Chat and faculty separately, a cumulative comparison was conducted to evaluate differences in grading for each question. Question III (QIII) specifically addressed whether discrepancies existed between Bing Chat and faculty assessments based on the type of question, as categorized in Table 1. ​This analysis aimed to identify patterns or inconsistencies in grading that may be influenced by question format or content, offering insight into how each evaluator responds to different categories of questions.

**Figure 3:**
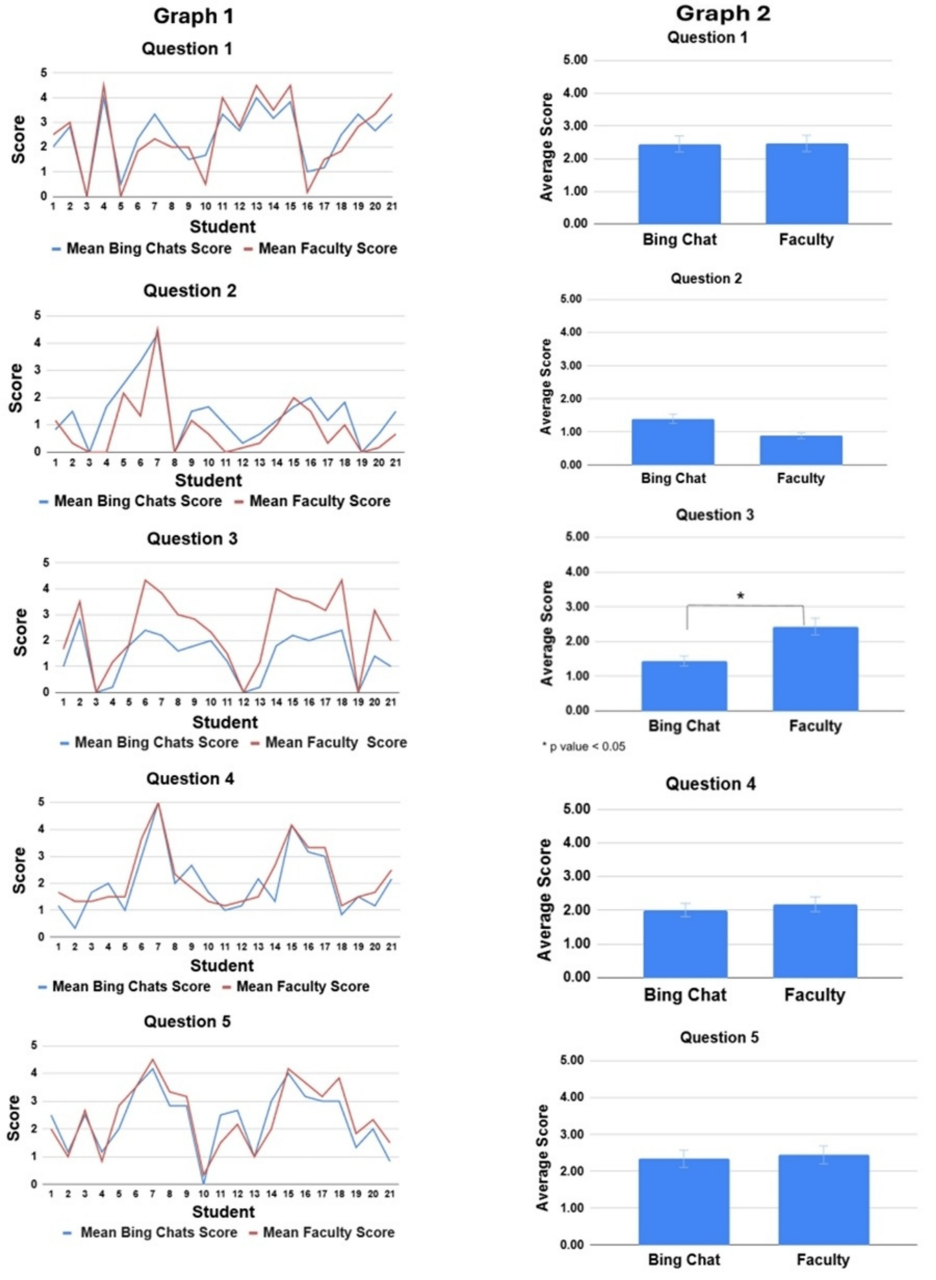
The graphs on the left correspond to graph 1 of questions 1 through 5, while the graphs on the right represent graph 2 for the same questions. The line graphs depict the averages and trends in scoring for each individual response, as assessed by Bing Chat and faculty. The bar graphs show the average scores assigned by Bing Chat and faculty for all 21 responses combined for each individual question.

In Table 1, question 1 falls into the question domain of analysis; question 2 is categorized as a problem-solving question with elements of application; question 3 involves recall and application; question 4 is explanatory; and question 5 is classified as a recall-only question. The average scores assigned by six Bing Chat accounts and six faculty members for each of the 21 individual student responses were calculated. The averages and trends in scoring for each individual response by each group can be seen in the line graph (Figure 3, graph 1). Additionally, the average scores assigned by Bing Chat and faculty for all 21 responses combined, per question, are presented in the bar graph (Figure 3, graph 2). These averages were used to compare, from a broader, simplified perspective, the differences between Bing Chat and faculty grading.

Overall, with the exception of question 3, Bing Chat and faculty scoring for individual responses closely follow each other, as seen in graph 1 (line graphs) for questions 1 through 5. Graph 1 of question 3 shows more variance between Bing Chat and faculty scoring for individual responses (Figure 3, graph 1 of question 3). Furthermore, Bing Chat and faculty exhibit similarity in the average scores across all the responses combined for each question, as shown in graph 2 (right bar graphs) of questions 1 through 5 - again with the exception of graph 2 of question 3. Graph 2 of question 3 demonstrates a statistically significant difference between Bing Chat and faculty grading (p = 0.010) (Table 8).

**Table 8:** Average scores for each individual, as assessed per question by six Bing Chat users and six faculty members per question. Q1-Q5: Question 1 through 5; C: Bing Chat; F: faculty; SD: standard deviation; CI: confidence interval.

	Q1		Q2		Q3		Q4		Q5	
	C	F	C	F	C	F	C	F	C	F
Mean ± SD	2.450 ± 1.138	2.470 ± 1.475	1.400 ± 1.073	0.880 ± 1.065	1.440 ± 0.900	2.430 ± 1.414	2.010 ± 1.155	2.180 ± 1.108	2.340 ± 1.087	2.440 ± 1.175
p-value	0.961		0.123		0.010		0.629		0.776	
95% CI	-0.842 to 0.802		-0.147 to 1.187		-1.729 to -0.251		-0.876 to 0.536		-0.806 to 0.606	

**Figure 4:**
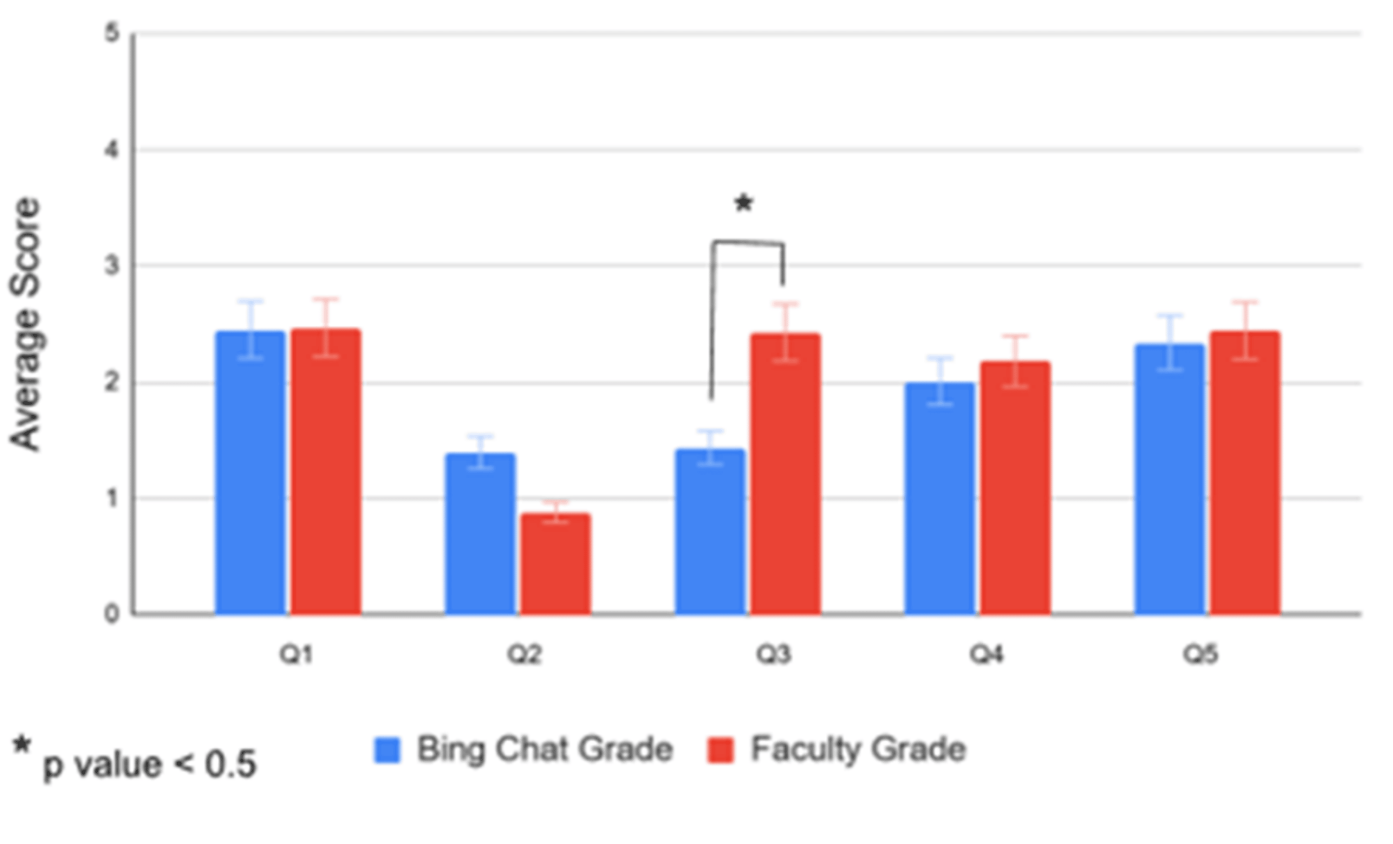
Mean Bing Chat and faculty scores. A consolidation of the bar graphs of graph 2 of questions 1 through 5 in Figure 3. A statistically significant difference exists between Bing Chat scores and faculty scores for question 3 (p = 0.010). Q1-Q5: Question 1 through 5.

For better illustration, the bar graph in Figure 4 shows the mean and the standard deviation for all five questions combined. It essentially consolidates the bar graphs of graph 2 of questions 1 through 5 in Figure 3. The purpose of these averages was to compare, from a broader, simplified perspective, the differences between Bing Chat and faculty grading. Overall, the average scores are relatively similar, with question 3 being the only one that shows a statistically significant difference (p = 0.010) (Table 8).

**Figure 5:**
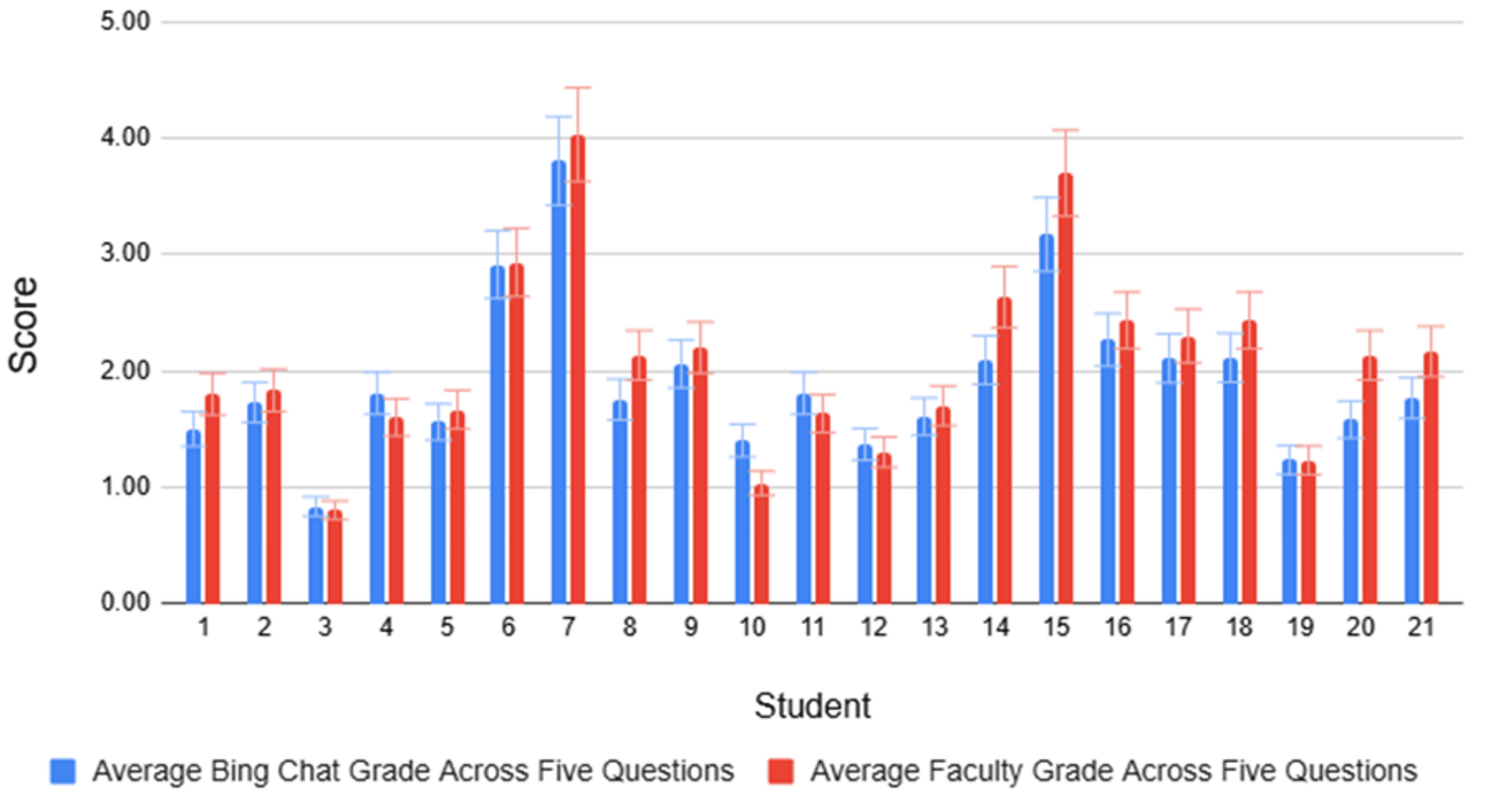
Comparison between Bing Chat and faculty grading for all 21 student responses across all five questions. Overall, there is no statistically significant difference between Bing Chat and faculty scores.

To obtain a broader perspective on potential variation in scoring, the average of Bing Chat and faculty scores for each student across all five questions was calculated and graphed. This analysis addressed question IV (QIV): "How different are the grades assigned by Bing Chat versus faculty for each respondent across all five questions?"​ An unpaired t-test was used to determine whether there is a statistically significant difference between the averages of the two groups. Figure 5 compares the average scores between Bing Chat and faculty grading for all responses across all five questions. In this case, there is no statistically significant difference between Bing Chat and faculty grading (0.437 ≤ p ≤ 1.000) (Table 9).

**Table 9:** Summary statistics for Bing Chat and faculty scores of 21 student responses across five questions. SD: standard deviation; CI: confidence interval.

	1	2	3	4	5	6	7	8	9	10	11	12	13	14	15	16	17	18	19	20	21
Average Bing Chat grade across five questions	1.500	1.730	0.830	1.810	1.560	2.910	3.810	1.750	2.060	1.400	1.810	1.370	1.610	2.090	3.170	2.270	2.110	2.110	1.230	1.580	1.770
Bing Chat SD	0.720	1.080	1.180	1.400	0.800	0.530	1.080	1.080	0.640	0.800	1.060	1.260	1.520	0.930	1.150	0.920	0.920	0.830	1.370	0.770	1.020
Average faculty grade across five questions	1.800	1.830	0.800	1.600	1.670	2.930	4.030	2.130	2.200	1.030	1.630	1.300	1.700	2.630	3.700	2.430	2.300	2.430	1.230	2.130	2.170
Faculty SD	0.490	1.350	1.190	1.710	1.050	1.280	1.040	1.300	0.800	0.820	1.460	1.230	1.620	1.190	1.000	1.540	1.330	1.550	1.230	1.290	1.310
p-value	0.463	0.900	0.969	0.837	0.857	0.975	0.751	0.629	0.768	0.491	0.829	0.931	0.930	0.447	0.459	0.847	0.799	0.695	1.000	0.437	0.605
95% CI	-1.198 to 0.598	-1.883 to 1.683	-1.698 to 1.758	-2.069 to 2.489	-1.471 to 1.251	-1.449 to 1.409	-1.766 to 1.326	-2.123 to 1.363	-1.197 to 0.917	-0.811 to 1.551	-1.681 to 2.041	-1.746 to 1.886	-2.381 to 2.201	-2.098 to 1.018	-2.102 to 1.042	-2.010 to 1.690	-1.858 to 1.478	-2.133 to 1.493	-1.899 to 1.899	-2.099 to 0.999	-2.112 to 1.312

Inter-rater reliability results

The objective of this analysis was to compare the inter-rater agreement between Bing Chat and faculty in grading 21 responses to five questions using two methods: percent agreement and Gwet's AC1. The five questions, answered by 21 participants, were presented to and graded by each group (Bing Chat and faculty group) following the same grading rubric. Percent agreement and Gwet's AC1 coefficients are kappa measurements, which measure agreement or reliability between raters [11,15]. Possible values for kappa statistics range from -1 to 1, with 1 indicating perfect agreement, 0 indicating completely random agreement, and -1 indicating perfect disagreement [11,15]. According to Landis and Koch (1977), the guidelines for interpreting kappa values or coefficient values are as follows: values from 0.00 to 0.20 indicate slight agreement, 0.21 to 0.40 indicate fair agreement, 0.41 to 0.60 indicate moderate agreement, 0.61 to 0.80 indicate substantial agreement, and 0.81 to 1.0 indicate almost perfect or perfect agreement [15].

**Figure 6:**
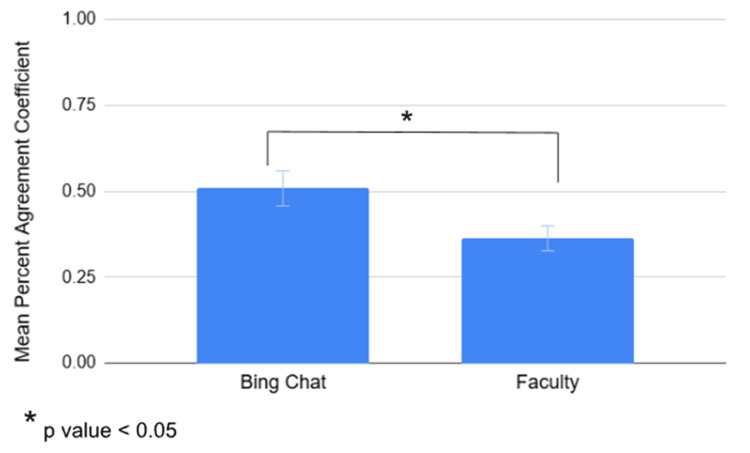
Mean percent agreement coefficients for the Bing Chat group and the faculty group, based on inter-rater reliability coefficients calculated from scores assigned by six Bing Chat users and six faculty members for each of 21 students across five questions. A statistically significant difference in mean percent agreement coefficients was observed between Bing Chat and faculty when examined across five questions (p = 0.0005).

**Figure 7:**
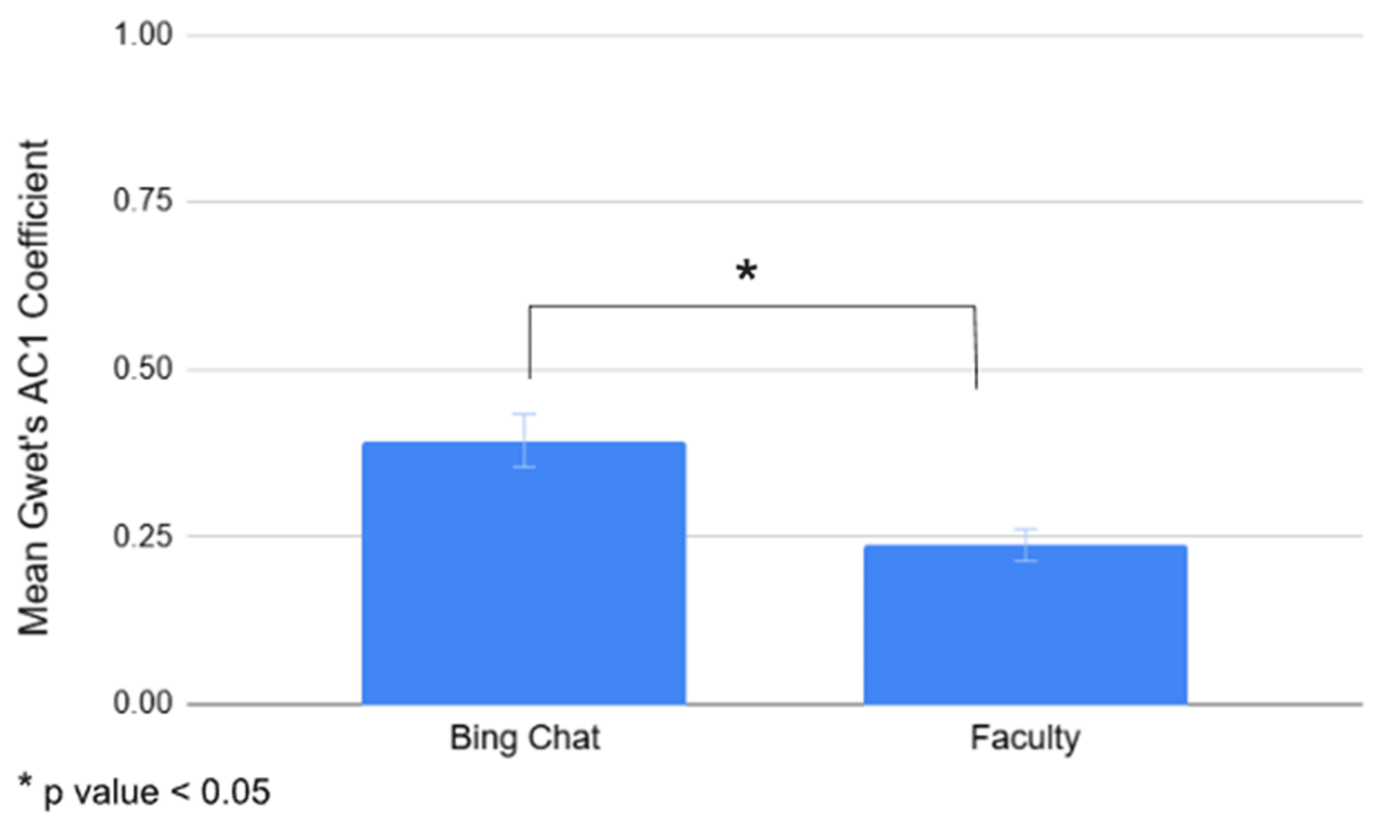
Mean Gwet's agreement coefficients for the Bing Chat group and faculty group for 21 students across five questions. The coefficient of the Bing Chat group was higher than that of the faculty group. A statistically significant difference in mean Gwet's agreement coefficients was observed between Bing Chat and faculty when examined across five questions (p = 0.0018).

The mean percent agreement coefficient was calculated with the percent agreement coefficients from six raters (either six Bing Chat accounts or six faculty members) for each of 21 students across five questions. The Bing Chat group yielded a mean percent agreement coefficient of 0.5085, indicating moderate agreement among Bing Chat raters (Table 10). In contrast, the faculty group had a mean percent agreement coefficient of 0.3631, reflecting fair agreement among faculty members (Table 10). The mean percent agreement coefficient for Bing Chat was higher than that of the faculty, and this difference in inter-rater reliability across five questions was statistically significant (p = 0.0005) (Figure 6). Using a similar approach, the mean Gwet's agreement coefficient was calculated for each group. The mean Gwet's agreement coefficient for Bing Chat was 0.3938, indicating fair agreement among Bing Chat raters, while the faculty's group mean coefficient was 0.2378, also indicating fair agreement among faculty members (Table 10). Again, Bing Chat's Gwet’s mean agreement coefficient was higher than that of the faculty, and the difference was statistically significant (p = 0.0018) (Figure 7).

**Table 10:** Mean inter-rater reliability of percent agreement and Gwet's agreement coefficients of 21 students across five questions. CI: confidence interval; SD: standard deviation.

	Mean ± SD, Percent agreement coefficient	Mean ± Gwet's agreement coefficient
Bing Chat	0.5085 ± 0.1391	0.3938 ± 0.1703
Faculty	0.3631 ± 0.1060	0.2378 ± 0.1295
p-value	0.0005	0.0018
95% CI	0.0683 to 0.2225	0.0616 to 0.2504

**Figure 8:**
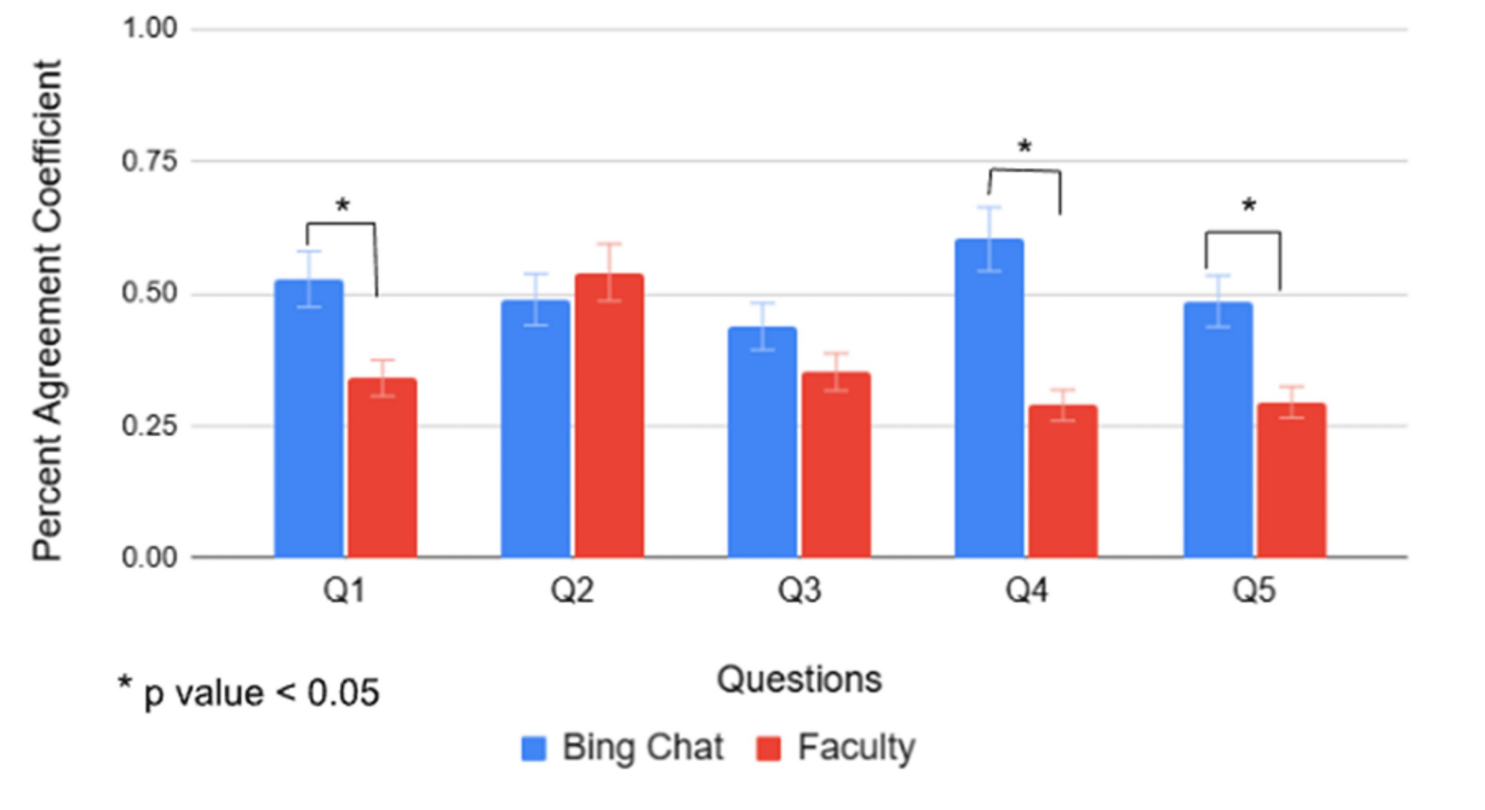
Percent agreement coefficients of Bing Chat and faculty, compared by question. Statistically significant differences were observed between Bing Chat and faculty for question 1 (p ≈ 0.031), question 4 (p ≈ 1.0 × 10⁻⁵), and question 5 (p ≈ 1.5 × 10⁻⁴). Q1-Q5: Question 1 through 5.

Moreover, inter-rater reliability coefficients for each question were examined across 21 student responses, based on scores assigned by six Bing Chat users and six faculty members. Figure 8 shows the mean percent agreement coefficients obtained from Bing Chat and faculty raters for questions 1 through 5. Slight differences in mean percent agreement coefficients between Bing Chat and faculty were observed for question 2 and question 3, but no statistically significant differences were found. In contrast, statistically significant differences in percent agreement coefficients were found for question 1 (p ≈ 0.031), question 4 (p ≈ 1.0 × 10⁻⁵), and question 5 (p ≈ 1.5 × 10⁻⁴) (Figure 8). Bing Chat percent agreement coefficients for questions 1, 4, and 5 were 0.527, 0.603, and 0.486, respectively, all falling within the moderate agreement (Table 11). In comparison, faculty percent agreement coefficients in questions 1, 4, and 5 were 0.340, 0.289, and 0.295, respectively, indicating a fair agreement among faculty members (Table 11).

**Table 11:** Mean inter-rater reliability percent agreement and Gwet's agreement coefficient 1 (AC1) on each question for 21 students. P: percent agreement coefficient; G: Gwet's agreement coefficient; SE: standard error.

	Question									
	1		2		3		4		5	
	P	G	P	G	P	G	P	G	P	G
Bing Chat coefficient (SE)	0.527 (0.057)	0.416 (0.071)	0.489 (0.054)	0.401 (0.064)	0.440 (0.065)	0.311 (0.08)	0.603 (0.052)	0.535 (0.062)	0.486 (0.041)	0.393 (0.047)
Faculty coefficient (SE)	0.340 (0.065)	0.209 (0.079)	0.540 (0.068)	0.473 (0.085)	0.352 (0.058)	0.250 (0.068)	0.289 (0.041)	0.161 (0.048)	0.295 (0.029)	0.158 (0.034)
p-value	0.031	5.450E-02	0.558	2.290E-01	0.312	7.310E-01	1.000E-05	9.830E-05	1.500E-04	2.930E-05

**Figure 9:**
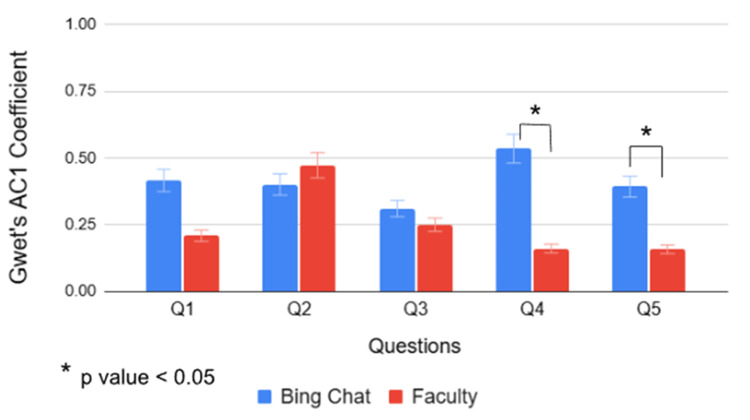
Inter-rater reliability of Bing Chat and faculty by question using Gwet's agreement coefficient 1 (AC1) method. Statistically significant differences in Gwet’s agreement coefficients were observed between Bing Chat and faculty for question 4 (p = 9.830 × 10⁻⁵) and question 5 (p = 2.930 × 10⁻⁵). Q1-Q5: Question 1 through 5.

Figure 9 shows the inter-rater reliability of Bing Chat and faculty, based on scores each group assigned to 21 students per question using Gwet's AC1 method. As with percent agreement, there were slight differences in Gwet's agreement coefficients between Bing Chat and faculty for questions 2 and 3 and a more pronounced difference in question 1; however, these differences were not statistically significant. In contrast, statistically significant differences in Gwet's agreement coefficients were observed for questions 4 (p = 9.830 × 10⁻⁵) and question 5 (p = 2.930 × 10⁻⁵) (Figure 9). Bing Chat Gwet's agreement coefficients for questions 4 and 5 were 0.535 (moderate agreement) and 0.393 (fair agreement), respectively (Table 11). In comparison, faculty's Gwet's agreement coefficients for questions 4 and 5 were 0.161 and 0.158, respectively, indicating slight agreement among faculty members (Table 11).

## Discussion

The primary objective of this study was to evaluate the effectiveness of Bing Chat in grading open-ended responses. Regarding whether different Bing Chat accounts consistently assign similar scores to the same answer (QI), the overall results show consistency, with only slight variations among Bing Chat accounts. This study also addresses whether grading by faculty members for the same response is consistent (QII). Variations were observed among the faculty members when grading the same responses, which appeared more prominent than those among Bing Chat accounts. Faculty subjectivity may have contributed to the variability seen in faculty scores.

In addition, QIII investigates whether the type of question influences grading differences between the two groups. Overall, except for question 3, Bing Chat and faculty scores for individual responses closely follow each other. However, there is more variance between Bing Chat and faculty scores for individual responses in question 3, and this difference was statistically significant. While question 1 falls into the category of analysis, question 2 into problem-solving with elements of application, question 4 is explanatory, and question 5 involves simple recall, question 3 combines recall and application. In question 3, students were asked to provide the correct anatomic sequence of vessels traversed by a Swan-Ganz catheter if introduced into the left subclavian vein. This requires the students to know what a Swan-Ganz catheter is, as hinted at in the vignette. Interestingly, Bing Chat scores deviated significantly from those of the faculty. Although this experiment found less variance in scoring between Bing Chat accounts compared to faculty members, Bing Chat assigned lower scores than the faculty for question 3. The higher scores given by faculty may reflect subjectivity in grading, whereas Bing Chat applies the rubric more stringently and is less affected by subjectivity. Further research is recommended to explore this fully. No significant differences were observed across the domains of analysis, problem-solving with elements of application, explanatory, and recall. In addition, QIV examines whether the average scores assigned by Bing Chat and faculty for each respondent across all five questions differ. Overall, no statistically significant difference was found between Bing Chat and faculty when grading for all responses across the five questions (0.437 ≤ p ≤ 1.000), indicating that the two grading methods are comparable. Both Bing Chat and faculty measure the same response with consistency, albeit with some variability in both, but more so in the faculty's grading. A key takeaway is that Bing Chat demonstrated more consistency than faculty when grading the same student response, was less affected by subjectivity, and adhered more strictly to the rubric.

For further analysis, an inter-rater reliability study was conducted to compare Bing Chat and faculty grading of 21 responses to five questions, using percent agreement and Gwet's AC1 coefficients. When comparing the mean percent agreement coefficients obtained from six raters (either six Bing Chat accounts or six faculty members) for each student across five questions, the Bing Chat group displayed moderate agreement (0.5085), compared to the faculty group's fair agreement (0.3631). It can be concluded that the Bing Chat group showed higher agreement than the faculty group when percent agreement coefficients were used to assess each student’s responses across five questions; this difference is statistically significant (p = 0.0005). Using a similar approach, the mean Gwet's agreement coefficient was calculated for each group. Both the Bing Chat group (0.3938) and the faculty group (0.2378) displayed fair agreement. Although both groups fell within the same agreement category, the Bing Chat group had a higher Gwet's agreement coefficient than the faculty group, and this difference again was statistically significant (p = 0.0018). Overall, Bing Chat demonstrated greater inter-rater reliability than faculty when grading each student's responses across five questions.

Additionally, when examining inter-rater reliability of Bing Chat and faculty for each question answered by 21 students using percent agreement, statistically significant differences were found for question 1 (p ≈0.031), question 4 (p ≈ 1.0 × 10⁻⁵), and question 5 (p ≈ 1.5 × 10⁻⁴). The Bing Chat group's percent agreement coefficients for questions 3, 4, and 5 were 0.527, 0.603, and 0.486, respectively, all falling within the moderate agreement scale. In comparison, the faculty group’s percent agreement coefficients for questions 3, 4, and 5 were 0.340, 0.289, and 0.295, respectively, indicating only fair agreement among faculty members. These findings suggest that Bing Chat demonstrates stronger inter-rater reliability than faculty for analysis-, explanatory-, and recall-type questions when measured using percent agreement for each question across 21 responses. When question types are combined, as in question 2 (problem-solving with elements of application) and question 3 (recall and application), Bing Chat demonstrated moderate agreement for both (0.489 and 0.440, respectively), while faculty displayed moderate (0.540) and fair (0.352) agreement, respectively. The slight differences observed between Bing Chat and the faculty’s percent agreement coefficients for questions 2 and 3 were not statistically significant. Thus, it can be concluded that when evaluating combination-type questions, Bing Chat and faculty performed comparably.

Similarly, when examining the inter-rater reliability of Bing Chat and faculty based on scores each group provided to 21 students per question using Gwet's AC1 method, statistically significant differences were also noted for questions 4 (p = 9.830 × 10⁻⁵) and 5 (p = 2.930 × 10⁻²). Bing Chat demonstrated moderate agreement for question 4 (0.535) and fair agreement for question 5 (0.393). In comparison, faculty’s Gwet's agreement coefficients for questions 4 and 5 were 0.161 and 0.152, respectively, indicating only slight agreement among faculty members. No statistically significant differences were observed between Bing Chat and the faculty’s Gwet's AC1 coefficients for questions 1, 2, and 3. Notably, question 1, previously showing a statistically significant difference in percent agreement coefficients between Bing Chat and faculty on scores assigned to 21 students per question, now yielded a marginally non-significant result (p = 0.0545) when using Gwet's AC1. This discrepancy may be attributed to Gwet's accounting for chance agreement, whereas percent agreement does not [11,16].

Regarding the statistically significant differences found between Bing Chat and the faculty’s inter-rater reliability for questions 1, 4, and 5 when measured using percent agreement and Gwet's AC1, a marginal non-statistical difference for question 1, and statistical differences for questions 4 and 5 are worth noting. In these cases, Bing Chat demonstrated higher agreement coefficients than faculty members, indicating higher inter-rater reliability among Bing Chat user accounts. These elevated agreement coefficients among the Bing Chat group may be attributed to the nature of the questions, which were non-combined in type. Question 1, an analysis-type question, provided students with sufficient hints from the vignette to deduce the type of cells used in the experiment. Question 4 ("What is the physiological cause of red cell sickling in sickle cell disease?") was an explanatory-type question; question 5 ("Write down at least three notable signs or symptoms classically associated with polycythemia vera?") was a recall-only question (Table 1). All three are non-combined question types, which tend to have more straightforward answers and are less complicated to grade. Also, the notion that Bing Chat is less influenced by subjectivity may have contributed to its higher scores. The low agreement coefficients among faculty members for questions 1, 4, and 5 may be due to subjectivity in grading, as well as a lack of coherence in rubric interpretation or in topic understanding, which may have contributed to this discrepancy.

There are several limitations to this study. To begin with, Bing Chat's scoring in this study does not account for different locations or times. Bing Chat scoring may have been influenced by these factors, which were not controlled for in this study. Secondly, in some studies, the same task was repeated two or three times to the same Bing Chat account, as repeated requests of the same tasks can yield different results [17,18]. Intra-rater reliability test was not accounted for in this study. Additionally, only one question was included per question domain. Including two or more questions within each domain would have strengthened the study design. This may be considered as the next step in this study to determine whether similar results are observed across multiple questions within the same question domain. Increasing the sample size to improve response rates through greater incentives is an additional consideration. Lastly, it may be necessary to conduct grading calibration sessions with faculty, provide training in relevant topics, or select faculty with a strong-matter expertise to promote consistency and agreement across faculty graders.

There are risks and challenges associated with using Bing Chat or similar AI tools for grading or evaluating exams, particularly open-ended questions. AI cannot fully understand nuance, creativity, or context in student answers that humans appreciate and may penalize unconventional but correct reasoning. As noted in this study, Bing Chat adheres strictly to rubrics, which can result in lower scores for answers that faculty might grade more leniently. A need to further investigate this exists. Furthermore, ethical and privacy concerns arise when using AI for grading, particularly regarding data security and transparency in assessment. It is imperative that any identifying information that could reveal a student’s identity be concealed. Additionally, consent from the students and transparency regarding the use of Bing Chat or other AI tools for evaluating their responses should be obtained. In this study, no identifying information was entered into Bing Chat; students were informed, and consent was obtained. Students may also question the fairness or accuracy if they know an AI graded their work. In this study, although limited by a small sample size and the number of questions, Bing Chat scores appeared more consistent than those of the faculty and, for the most part, aligned with faculty scoring, except in the combined recall and application question domain when evaluating responses.

When benchmarked against faculty, Bing Chat shows promise in grading written responses for open-ended questions and has the potential to perform effectively as an assistive grading tool. Bing Chat is making a considerable contribution to the field of education, with administrators on the lookout for grading solutions that are more dependable, efficient, and economical. Learners and educators can both benefit from Bing Chat. For learners, it can enhance long-term retention of medical knowledge by facilitating active recall through answering open-ended questions and receiving rapid, targeted feedback responses generated from Bing Chat. This dynamic interaction can help students identify and address their areas of weakness more effectively. For educators, Bing Chat offers relief from administrative burdens by automating routine tasks and streamlining workflows, requiring minimal training for implementation. This allows educators to focus more time on refining their lesson content and optimizing their delivery methods. This research builds on existing efforts to explore how large language models (LLMs) such as Bing Chat can enhance the educational experience for students and alleviate educators' administrative burdens.

## Conclusions

In this study, Bing Chat demonstrated consistency than faculty when grading identical student responses. While overall grading patterns were comparable, Bing Chat assigned lower scores than faculty on a combined recall-and-application question, likely due to its stricter adherence to the rubric. In contrast, faculty scores may have reflected subjective judgment. Inter-rater reliability analysis, using percent agreement and Gwet’s AC1, showed that Bing Chat generally achieved higher agreement levels, particularly in non-combined question types. These findings suggest that Bing Chat, with its reduced susceptibility to subjective bias, has the potential to provide consistent, timely feedback and serve as a valuable tool for supporting both faculty and students. However, these results are preliminary and should be interpreted with caution, as further research is needed to confirm their role in educational assessment.

## Appendices

Appendix A

Questions With Expert Answers

1. A cell biologist is interested in the role of ribonucleoproteins in cellular processes. He isolates a specific type of cells from a human female donor who is 60 years old and extracts the ribonucleoproteins from them. He then performs structural and functional analyses on the purified ribonucleoproteins. He discovers that these cells have a higher expression of a protein that can synthesize TTAGGG repeats at the ends of chromosomes using reverse transcription. Explain what kind of cells he most likely used in his experiment and why?

Expert answer: Telomerase is a ribonucleoprotein that adds TTAGGG repeats to the 3’ end of chromosomes (telomere region). Stem cells have very long telomeres due to their high telomerase activity, allowing them to proliferate indefinitely in a controlled manner. In contrast, most terminally differentiated adult somatic cells (e.g., myocardial cells, neurons, pancreatic β cells) have short telomeres as they do not express telomerase and their telomeres shorten with every cell division. The epidermis is continuously replaced by stem cells present in the basal cell layers. Bone marrow stem cells similarly replace peripheral red and white blood cells. Stem cells have long telomeres due to high telomerase activity, allowing them to proliferate indefinitely in a controlled manner.

2. A 40-year-old man presents to the emergency department complaining of severe shortness of breath. The breathlessness has been worsening over the past few years, and the patient reports growing tachypneic with mild exertion, and sometimes even at night. On examination, he has generalized edema, jugular venous distention, and hepatic distention. Cardiac examination shows a right ventricular heave, a right-sided S3, and S4 with a pulmonary ejection click. A chest X-ray film shows cardiomegaly and widening of the hilar vessels, including the pulmonary arteries. An electrocardiogram shows tall, peaked P waves in leads II, III, and aVF, right axis deviation, and right ventricular hypertrophy. Which physiologic stimuli will result in decreased pulmonary vascular resistance in this patient?

Expert answer: This patient has cor pulmonale, which is defined as enlargement of the right ventricle secondary to diseases of the lung, thorax, or pulmonary circulation. In this case, it is chronic, given the duration of the patient's symptoms and the presence of many clinical sequelae of the condition: edema, jugular venous distention, hepatic distention, and right ventricular heave. The electrocardiogram also supports the diagnosis of enlargement of the right ventricle, showing right axis deviation due to the increase in the mass of the right heart. Evidence of right atrial enlargement is also present, i.e., the tall, peaked P waves in leads II, III, and aVF (P pulmonale). A unique feature of the pulmonary circulation is that it maintains itself as a low-pressure system. Many of the mechanisms that control pulmonary vasculature differ from those of the systemic circulation. One of these features is that pulmonary vasculature resistance is decreased in response to increased cardiac output. This is accomplished through distention of open capillaries and the recruitment of collapsed capillaries. Thus, the resistance in the pulmonary tree decreases in response to increased right ventricular output.

3. Pulmonary hypertension is suspected in the 40-year-old patient with dyspnea, and a Swan-Ganz catheter is placed. Provide the correct anatomic sequence of vessels that would be traversed by the catheter if it were introduced into the left median cubital vein (start with the left median cubital vein)?

Expert answer: The correct sequence for a catheter inserted into the left subclavian vein is as follows: median cubital vein, basilic or cephalic vein, left subclavian vein, left brachiocephalic vein, superior vena cava, right atrium, right ventricle, pulmonary artery. With this catheter in place, a variety of cardiac parameters can be measured, including pressures in the pulmonary artery. Thus, this catheter can aid in establishing the diagnosis of pulmonary hypertension.

4. What is the physiological cause of red cell sickling in sickle cell disease?

Expert answer: The primary initiating event is caused by the polymerization of deoxygenated HbS. The hemoglobin in sickle cell disease (HbS) becomes insoluble, especially in states of low oxygen, and can precipitate, deforming and harming the RBC. Conditions that promote sickling of RBCs are the same conditions that shift the oxygen dissociation curve to the right, including elevated temperature (fever), hypercapnia, hypoxia, low-flow vessels (where hemoglobin has the time to release lots of oxygen), and acidosis (which decreases hemoglobin affinity for oxygen). In general, any type of systemic infection (including common colds) may also precipitate sickling.

5. Write down at least three notable signs or symptoms classically associated with polycythemia vera.

Expert answer: Clinical manifestations of polycythemia vera include: aquagenic pruritus (itching following exposure to warm water); erythromelalgia (episodic blockage of blood vessels followed by burning pain in the extremities with erythema and cyanosis); symptomatic gout (5-10% of cases); splenomegaly; blurry vision/headache; facial plethora (flushing).

Appendix B

Example prompt:

“Please provide a concise response to the question/prompt below. Your answer will be graded based on accuracy and completeness. Use the following scale to assess each response:

5: The answer is complete. All information provided is accurate.

4: The answer is missing two or fewer details. All information provided is accurate.

3: The answer is missing multiple (>2) details. Most information provided is accurate.

2: Content suggests a lack of preparation or comprehension. Some information provided is accurate.

1: Content is only marginally related to the question/prompt. A small amount of the information is accurate.

0: Content fails to meet the basic requirements of the task. None of the information provided is accurate.”

Below is the question we are going to be testing with the best answer provided (given a grade of 5 of 5):

A cell biologist is interested in the role of ribonucleoproteins in cellular processes. He isolates a specific type of cells from a human female donor who is 60 years old and extracts the ribonucleoproteins from them. He then performs structural and functional analyses on the purified ribonucleoproteins. He discovers that these cells have a higher expression of a protein that can synthesize TTAGGG repeats at the ends of chromosomes using reverse transcription. Explain what kind of cells he most likely used in his experiment and why?

Expert answer: Telomerase is a ribonucleoprotein that adds TTAGGG repeats to the 3’ end of chromosomes (telomere region). Stem cells have very long telomeres due to their high telomerase activity, allowing them to proliferate indefinitely in a controlled manner. In contrast, most terminally differentiated adult somatic cells (e.g., myocardial cells, neurons, pancreatic ß cells) have short telomeres as they do not express telomerase and their telomeres shorten with every cell division. The epidermis is continuously replaced by stem cells present in the basal cell layers. Bone marrow stem cells similarly replace peripheral red and white blood cells. Stem cells have long telomeres due to high telomerase activity, allowing them to proliferate indefinitely in a controlled manner.

Grade the following student response based on the grading criteria and the expert answer:

Student 1 response to question 1: “TTAGGG is associated with the repeating sequences that compose telomeres, which prevent loss of genetic material over time. I can’t recall what cell is involved in telomerase activity, but I think it’s that”.

Appendix C

Questionnaire

Revolutionizing Educational Assessment: Bing Chat's Role in Grading of Open-Ended Questions Survey

Dear Participant, 

Utilizing open-ended questions in medical education has significant benefits, as these questions go beyond testing knowledge and also help develop higher cognitive levels, such as analysis, synthesis skills, and problem-solving ability in future doctors. Open-ended questions make it possible to assess the ability to organize thoughts for different topics, so the learner needs a deep understanding to answer these questions. Due to the subjective nature of assessing answers for open-ended questions, errors in grading answers are possible. This is our pilot study on utilizing Bing Chat in the domain of open-ended question grading to reduce the subjectivity of grading open-ended questions. To achieve our goal, we are asking you to answer a set of FIVE open-ended questions that cover high-yield selected topics of basic sciences. All responses are entirely anonymous, and none of your answers can be linked to you in any way. You will not receive a grade for your individual work, but the correct answers will be available via the link at the end of the survey. It will take approximately 10-15 minutes to complete this survey.

You have a chance to win a $100 USD gift certificate by clicking the link that will appear after you finish answering the questions. This step is optional. The raffle winners will be announced between October 30th and November 15th, 2023.

Thank you very much for your cooperation!!!

Participating in this study is entirely optional, and you are able to stop at any time without penalty to you. All responses are entirely anonymous, and none of your answers can be linked to you in any way.

Do you consent to participate in this research?

• Yes • No

During this portion of the study, you will be asked to answer open-response questions relating to high-yield Step 1 topics. Attempt to answer these questions the best you can without any outside resources (Google/notes/friends). Please do not use copy and paste!!!! Even if you do not recognize a question, please make sure not to leave any section blank, N/A, etc. Please try to write a complete answer, even if you know it is wrong. Spelling and grammar will not be considered in the grading of each answer. Please limit your answers to no more than 200 words, if possible. You will not receive a grade for your individual work, but the correct answers will be posted at this link (please bookmark this link): https://docs.google.com/document/d/1yXYWtShcZk9QbQEBjax9XeMg667ZNDVBfMe5Uy8q49U/edit?usp=sharing by October 30th, 2023.

1. A cell biologist is interested in the role of ribonucleoproteins in cellular processes. He isolates a specific type of cells from a human female donor who is 60 years old and extracts the ribonucleoproteins from them. He then performs structural and functional analyses on the purified ribonucleoproteins. He discovers that these cells have a higher expression of a protein that can synthesize TTAGGG repeats at the ends of chromosomes using reverse transcription. Explain what kind of cells he most likely used in his experiment and why?

2. A 40-year-old man presents to the emergency department complaining of severe shortness of breath. The breathlessness has been worsening over the past few years, and the patient reports growing tachypneic with mild exertion, and sometimes even at night. On examination, he has generalized edema, jugular venous distention, and hepatic distention. Cardiac examination shows a right ventricular heave, a right-sided S3, and S4 with a pulmonary ejection click. A chest X-ray film shows cardiomegaly and widening of the hilar vessels, including the pulmonary arteries. An electrocardiogram shows tall, peaked P waves in leads lI, III, and aVF, right axis deviation, and right ventricular hypertrophy. Which physiologic stimuli will result in decreased pulmonary vascular resistance in this patient?

3. Pulmonary hypertension is suspected in the 40-year-old patient with dyspnea, and a Swan-Ganz catheter is placed. Provide the correct anatomic sequence of vessels that would be traversed by the catheter if it were introduced into the left median cubital vein (start with the left median cubital vein)?

4. What is the physiological cause of red cell sickling in sickle cell disease?

5. Write down at least three most notable signs or symptoms classically associated with polycythemia vera.

What is your semester?

• First • Second • Third • Fourth • Fifth • Prefer not to answer

If you would like to be entered into a drawing for a $100 USD gift certificate, please click the link below to provide your information. This section is entirely optional and will not affect your participation in this study.

https://docs.google.com/forms/d/e/1FAIpQLSdK-nqjT5-1Znk7cu7TOI01zsiF9x7MYnOlXCjqMdsCs1F9fw/viewform?usp=sf_link

Please let us know your thoughts on how to improve our study (optional).
